# COM6 /485: Dermis.net - from a Dermatology Internet Atlas to a Dermatology Internet Portal Site

**DOI:** 10.2196/jmir.1.suppl1.e19

**Published:** 1999-09-19

**Authors:** G Eysenbach, J Paessler, TL Diepgen

**Affiliations:** ^1^Institute for Clinical Social Medicine, HeidelbergGermany

**Keywords:** Portal sites, Dermatology

## Abstract

**Introduction:**

To provide an image collection and entry point into dermatological resources on the World Wide Web, we have developed an image database (Dermatological Online Atlas--DOIA), which is now being transformed into a Internet portal site "Dermatology Information System" (DermIS), providing an entry point for dermatologists and consumers .

**Methods:**

The Web site runs on a WinNT Internet Server. Interactive components are programmed using Delphi, JAVA, JavaScript and Active Server Pages. Image information is stored in an Access database.

**Results:**

The database, which is freely available on the Internet at http://dermis.net, contains about 3,000 clinical images covering more than 600 dermatological diagnoses. It is designed for world-wide use; international submissions are encouraged. It serves as a teaching tool for medical students, doctors and patients, assists professional users by being an easy gateway to other databases such as MEDLINE, PDQ, and OMIM and serves as a platform for conducting research by means of administering Internet surveys to users. With more than 3000 distinct users per day, among them approximately 50% consumers, the Web site is one of the leading medical Web-sites in Germany.

**Discussion:**

With a further strengthening of textual information and interactive components, we envisage that the Online Atlas will become a universal starting point (portal site) for users seeking dermatological information on the Web.

